# Anti-adhesion and Anti-biofilm Potential of Organosilane Nanoparticles against Foodborne Pathogens

**DOI:** 10.3389/fmicb.2017.01295

**Published:** 2017-07-11

**Authors:** Eleni N. Gkana, Agapi I. Doulgeraki, Nikos G. Chorianopoulos, George-John E. Nychas

**Affiliations:** ^1^Laboratory of Microbiology and Biotechnology of Foods, Department of Food Science and Human Nutrition, Faculty of Foods, Biotechnology and Development, Agricultural University of Athens Athens, Greece; ^2^Institute of Technology of Agricultural Products, Hellenic Agricultural Organization-DEMETER Athens, Greece

**Keywords:** organosilanes, nanoparticles, biofilms, foodborne pathogens, anti-adhesion

## Abstract

Nowadays, modification of surfaces by nanoparticulate coatings is a simple process that may have applications in reducing the prevalence of bacterial cells both on medical devices and food processing surfaces. To this direction, biofilm biological cycle of *Salmonella* Typhimurium, *Listeria monocytogenes, Escherichia coli* O157:H7, *Staphylococcus aureus*, and *Yersinia enterocolitica* on stainless steel and glass surfaces, with or without nanocoating was monitored. To achieve this, four different commercial nanoparticle compounds (two for each surface) based on organo-functionalized silanes were selected. In total 10 strains of above species (two for each species) were selected to form biofilms on modified or not, stainless steel or glass surfaces, incubated at 37°C for 72 h. Biofilm population was enumerated by bead vortexing-plate counting method at four time intervals (3, 24, 48, and 72 h). Organosilane based products seemed to affect bacterial attachment on the inert surfaces and/or subsequent biofilm formation, but it was highly dependent on the species and material of surfaces involved. Specifically, reduced bacterial adhesion (at 3 h) of *Salmonella* and *E. coli* was observed (*P* < 0.05) in nanocoating glass surfaces in comparison with the control ones. Moreover, fewer *Salmonella* and *Yersinia* biofilm cells were enumerated on stainless steel coupons coated with organosilanes, than on non**-**coated surfaces at 24 h (*P* < 0.05). This study gives an insight to the efficacy of organosilanes based coatings against biofilm formation of foodborne pathogens, however, further studies are needed to better understand the impact of surface modification and the underlying mechanisms which are involved in this phenomenon.

## Introduction

During the last decades, it has become increasingly clear that biofilms are the predominant mode of bacterial growth in most of the natural environments (Lindsay and von Holy, 2006; Giaouris et al., 2013). Biofilm formation consists of at least two stages of development: the adherence of cells to an inert surface which may occur very rapidly and the formation of multilayered cell clusters surrounded by exopolysaccharides produced by bacteria (Götz, 2002). Initial adhesion process depends on bacterial species, interaction medium and inert surface (Pereni et al., 2006). Biofilm control or eradication occurs a considerable issue for food and medicine sector, since this complex bacterial community is resistant to antimicrobial and disinfectant agents (Hoyle and Costerton, 1991; Finlay and Falkow, 1997; Araújo et al., 2011; Bridier et al., 2011). Regarding the important medical and economic consequences of biofilm formation, the understanding of colonization process would be helpful in the design of surface modifications capable of preventing biofilm formation (Prigent-Combaret et al., 1999). Surface properties can be practically modified to reduce bacterial adhesion and further biofouling, which is a principal objective for food industries (Pereni et al., 2006). Surface modification refers to the alteration of physical and chemical properties of an inert substratum (roughness, hydrophobicity, etc.), leading to specific biochemical interactions that prevent bacterial attachment and thus biofilm formation (Kasimanickam et al., 2013).

Following this approach, nanomaterials were proposed as an interventional strategy for the management of biofilm formation due to their high surface area to volume ratio and unique chemical and physical properties (Morones et al., 2005). Nanomaterials were developed for a variety of food applications (food additives, food contact surfaces, food packaging, etc.) and for medical devices (catheter materials, dental acrylics, implants, etc.) (Harris and Graffagnini, 2007; Handford et al., 2014). Due to their small size (1–100 nm) and their ability to cover much larger surface to volume, they possessed altered physicochemical properties in comparison with larger sized material (Oberdörster et al., 2005; Bouwmeester et al., 2014). Nanoparticles such as ZnO (Heinlaan et al., 2008), TiO_2_ (Kim et al., 2003; Adams et al., 2006; Chorianopoulos et al., 2011) CuO (Heinlaan et al., 2008), and Al_3_O_2_ (Ansari et al., 2013). Compared to the quantum of published reports on physical and chemical properties of nanofilms, only limited information is available on the antibacterial properties of these nanomaterials.

Organo-functional silanes could be potential candidates for surface modifications, as can be used to modify the surface energy or wettability of substrates through the interaction of boundary layers of solids with water, effecting variable degrees of hydrophobicity or hydrophilicity (Mittal, 2009). Monomeric silicon chemicals are known as silanes and when they contain at least one silicon carbon bond (e.g., Si-CH_3_) are called organosilanes (Kregiel and Niedzielska, 2014). Organo-functional silanes are molecules carrying two different reactive groups on their silicon atom so that they can react with inorganic substrates such as glass and stainless steel and form stable covalent bonds and organic substitution (Thames and Panjnani, 1996; Sepeur, 2008). Several studies have examined the antimicrobial activity of nanoparticulate coatings constituted of silica and organosilanes; however, results retrieved are controversial.

Based on the above, the current study aimed to assess the potential anti-adhesion and anti-biofilm activity of commercial organosilane products applied on stainless steel and glass surfaces against common foodborne pathogens. To achieve this, biofilm biological cycle of *Salmonella* Typhimurium, *Listeria monocytogenes, Escherichia coli* O157:H7, *Staphylococcus aureus*, and *Yersinia enterocolitica* on stainless steel and glass surfaces, with or without nanocoating was monitored.

## Materials and Methods

### Bacterial Strains and Inocula Preparation

All the microorganisms used in this study are presented in **Table 1**. They consist of two strains of each species, specifically for *L. monocytogenes* (FMCC B-125, ScottA, serotype 4b, epidemic strain, human isolate; FMCC B-129, isolated from ready-to-eat frozen meal, minced meat based), *S.* Typhimurium (FMCC B-137, human isolate epidemic; FMCC B-193, isolated from calf bowel), *E. coli* O157:H7 (FMCC B-15 and FMCC B-16, both isolated from human feces), *S. aureus* [FMCC B-410, methicillin-resistant (MRSA) strain COL, isolated from hospital; FMCC B-135, isolated from human lesions], and *Y. enterocolitica* (FMCC B-89, CITY 650; FMCC B-90, CITY 844). Before each experiment the stock cultures (frozen at -80°C) were sub-cultured twice on 10 ml of Tryptic Soy Broth (TSB, LAB M Limited, Lancashire, United Kingdom) at 37°C for 24 and 16 h, respectively (pre-cultures). Cells from exponential phase (16 h) of cultures were collected by centrifugation (5000 × *g* for 10 min at 4°C), washed twice with 1/4 Ringer solution and re-suspended in 1/4 Ringer solution (working cultures) in order to be used as inoculum for biofilm assays.

**Table 1:** Bacterial species used in this study^∗^.

Microorganism	Strain number	Strain characteristics	Origin
*Listeria monocytogenes*	FMCC B-125	Scott A, Serotype 4b	Human isolated^a^
	FMCC B-129	21350	RTE frozen meal – minced meat based
*Salmonella* Typhimurium	FMCC B-137	DT 193 Multi-drug resistant	Human isolate epidemic^b^
	FMCC B-193	4/74	Isolated from calf bowel^c^
*E. coli* O157:H7	FMCC B-15	NCTC 13125, Verocytoxins negative	Human faeces^d^
	FMCC B-18	NCTC 13127, Verocytoxins negative	Human faeces^d^
*Staphylococcus aureus*	FMCC B-410	MRSA strain COL	English hospital^e^
	FMCC B-135	NCBF 1499	
*Yersinia enterocolitica*	FMCC B-89	CITY650	INCO^a^
	FMCC B-90	CITY844	INCO^a^

*^∗^ From bacterial culture collection of Laboratory of Microbiology and Biotechnology of Foods (FMCC), Agricultural University of Athens.*

*^a^ Kindly provided by Dr. E. Smid, ATO-DLO Netherlands.*

*^b^ Food Microbiology Culture Collection of Agricultural University of Athens.*

*^c^ Kindly provided by Dr. P. Skandamis.*

*^d^ Kindly provided by Dr. E. Drosinos.*

*^e^ Kindly provided by Dr. S. Kathariou, North Carolina State University, United States.*

### Biofilm Formation and Quantification on Polystyrene Microplates

The ability of 10 bacterial strains to form biofilms on polystyrene (PS) microtiter plates was evaluated by using the method described by Jena et al. (2012) with some adaptations. Working culture of above bacteria was diluted 1:100 into fresh medium TSB. Diluted culture (20 μl) was added to the 96-well plates containing 180 μl of TSB. The strains were grown in defined medium (TSB) at 37°C for 24 and 48 h in 96-wells microtiter plates under static conditions.

Following incubation, planktonic bacteria were removed by violently turning upside down the plate to remove growth medium and each well was then washed twice with 200 μl 1/4 Ringer solution to remove the loosely attached cells. The remaining adherent bacteria (biofilms) were fixed for 15 min with 200 μl of methanol per well (Stepanović, 2000). The methanol was discarded and the plates were left to air dry in room temperature for 20 min. Biofilm cells were stained with 100 μl of 1% Crystal Violet solution which was added at each well. After washing with 200 μl 1/4 Ringer three times to remove excess stain, the crystal violet was solubilized with 100 μl ethanol (95%) for 15 min. Dye absorbance at 575 nm (A575) was measured using a microtiter plate reader (Sunrise, Tecan, Männedorf, Switzerland). For each strain eight replicates were performed. Regarding the obtained spectrometric measurement of optical densities the strains were classified into the four categories; non-biofilm producing (OD <= 0.2), weakly (0.2 < OD <= 0.4), moderately (0.4 < OD <= 0.8), and strongly (0.8 < OD) biofilm producing strains according to the method proposed by Stepanović et al. (2004).

### Application of Commercial Organosilane Products for Modification of Stainless Steel and Glass Surfaces

Four organosilane based commercial products for coating of non-absorbing surfaces; two (2) for glass and two (2) for stainless steel, specific to each material surface according to manufacturers, were used. Specifically, three (3) commercial products that were obtained from Liquid Glass Nanotech^1^ with EINECS (European Inventory of Existing Commercial Chemical Substances) registration were used. The active agent was silicon-free siloxane and consists of polymers made of silanes. One (1) organosilane product for glass (OSG1) (Liquid Glass Nanotech for glass and ceramic surfaces, LGN-600-1) and two organosilane products for stainless steel (OSS1, OSS2) (Universal antimicrobial for non-absorbent/hard surfaces, LGN-671-ANTI; Polish for Metals and Plastics for non-absorbent/hard surfaces, LGN-660-1) were used. Moreover one (1) commercial organosilane based product for glass (OSG2) (NANO-SKIN [HOME]) from BFP Hellas Company^2^ was obtained, that is approved by General Chemical State Laboratory of Greece.

All the products were delivered as pump sprays for easy application and were applied following manufacturers’ instructions. Briefly, for products OSG1, OSS1, and OSS2 the application consisted of cleaning the surfaces with isopropyl alcohol and then rinsing with deionized water, spraying the coating on surface and evenly distribute the coating with a lint free microfiber cloth across the surface, polish off residue after 30 min and let the coating seal for at least 12–24 h. Nano-Skin product consists of three liquid mixtures (an emulsion and two sprays) which are applied sequentially. Pretreatment with emulsion NANO-SKIN (1) based on a specific composition, which restores the glass in its initial condition, was required. Then, NANO-SKIN (2) – an alcohol activating solution and NANO-SKIN (3) based on silicon oligomers, both sprayed subsequently to glass surface and spread with microfiber cloth, making gentle circular motions. All the aforementioned products sprayed onto a hard surface form a nano-film by self-organization during evaporation of the solvent (Sepeur, 2008). The film arises from the sol-gel process (Hench and West, 1990; Schmidt, 2006) that involves series of hydrolysis and condensation reactions between organo-functionalized silanes that result in a network of functionalized siloxanes (Nørgaard et al., 2014).

### Biofilm Formation on Stainless Steel and Glass Coupons

#### Preparation of Stainless Steel and Glass Surfaces

Stainless steel is the surface used extensively throughout the food processing industry. On the other hand glass was selected due to its high hydrophilicity and excellent silane effectiveness on this material. In addition, it is well known that significant portion of food deposits is made of glass (e.g., doors and coverings of refrigerators in super markets). Stainless steel is the surface used extensively throughout the food processing industry. Stainless steel (SS) coupons (3 by 1 by 0.1 cm, type AISI-304; Halyvourgiki, Inc., Athens, Greece) and glass (G) coupons (3 by 1 by 0.1 cm cut from microscope slides) were initially soaked in acetone (overnight) to remove any manufacturing process debris and grease. Coupons were then washed by soaking overnight at room temperature in a 2% (vol/vol) solution of the commercial detergent RBS 35 (Fluka/Life Science Chemilab, S.A.) with shaking, rinsed thoroughly with tap water followed by distilled water and air dried. The coupons were coated by the procedure mentioned above with commercial nano-coatings. Glass and stainless steel coupon without coating were used as control. Finally, cleaned coupons were individually placed in empty glass test tubes (length, 10 cm; diameter, 1.5 cm) and autoclaved at 121°C for 15 min.

#### Biofilm Formation and Enumeration

Ten strains (*S.* Typhimurium 137, 193, *S. aureus* 135, 410, *Y. enterocolitica* 89, 90, *E. coli* O157:H7 15, 18, and *L. monocytogenes* 125, 129) were selected to examine biofilm formation on stainless steel and glass surfaces, coated or not with organosilanes. Strains with different isolation origins (i.e., clinical, food, or environment) were selected in an attempt to pursue variability. The study was performed according to the protocol described by Kostaki et al. (2012) with minor modifications. The working cultures were diluted at 1:100 and 0.5 ml was added in 4.5 ml Ringer that contained a stainless steel or glass coupon. For the attachment step, 0.5 ml of each bacterial suspension in 4.5 ml quarter-strength Ringer solution, containing *ca.* 10^6^ CFU/ml, was poured into each glass test tube containing a sterilized coupon and incubated at 15°C for 3 h under static conditions. This temperature, representative of food industry during non-production hours (15°C) was incorporated in this study to investigate the adherent properties of abovementioned foodborne pathogens.

Following the attachment step, each coupon was carefully removed from the glass test tube using sterile forceps and individually introduced into a new sterile glass test tube containing 5 ml of TSB and subsequently incubated at 37°C for 3 days (72 h), under static conditions, to allow biofilm development on the coupon, with no growth medium renewal. Each experiment included three replications and sampling was performed at 3, 24, 48, and 72 h. A higher temperature (37°C) of incubation to determine biofilm formation was selected because previous studies have shown that biofilm production is increased when bacteria allowed growing next to or at their optimal temperature (Morton et al., 1998; da Silva Meira et al., 2012; Kadam et al., 2013). Furthermore, it was evaluated that at 37°C, *L. monocytogenes* biofilm exhibited a complex system, in terms of cell number and EPS produced, due to advanced state of growth rate. Therefore, this temperature (37°C) represents the worst-case scenario of biofilm formation in order to determine if there is a potential anti-biofilm activity of organosilanes.

Briefly, each coupon was aseptically removed from the glass test tube and was then rinsed by pipetting twice with 10 ml of quarter-strength Ringer solution (each time). The coupon was transferred to a falcon centrifuge tube containing 6 ml of quarter-strength Ringer solution and 10 sterile glass beads (diameter, 3 mm) and then vortexed for 2 min at maximum speed to detach biofilm cells from the coupon. Detached cells obtained by bead vortexing method (Giaouris and Nychas, 2006) were subsequently enumerated on Tryptone Soy Agar (TSA; Lab M), after 10-fold serial dilutions. Stainless steel and glass surfaces were examined under conventional fluorescence microscope using acridine orange stain to determine the absence of residual biofilm remained on substrate (data not shown).

#### Data Analysis

Univariate analysis of Variance (n-way ANOVA) for each stainless steel and glass surfaces was performed to test the main interaction effects of independent factors: (a) three different materials of surfaces (one non-coated and two coated surfaces), (b) five pathogen species (*S*. Typhimurium. *S. aureus, L. monocytogenes, Y. enterocolitica*, and *E. coli* O157:H7), and (c) four different time points (3, 24, 48, and 72 h) to bacterial attached cells as expressed by log CFU/cm^2^ (dependent). Thus, a 3^∗^5^∗^4 factorial design was constructed and when probability of F-values were less than 0.05 for any independent or combinations of independents, it was concluded that the variable has an effect on the depended. Each experiment was conducted using three replicates for each. The Tukey *post hoc* test was used to compare the means at the 95% confidence level. The statistical analysis was conducted using the IBM^®^ SPSS^®^ Statistics for Windows software, Version 22.0 (IBM Corp., Armonk, NY, United States).

## Results

**FIGURE 1:**
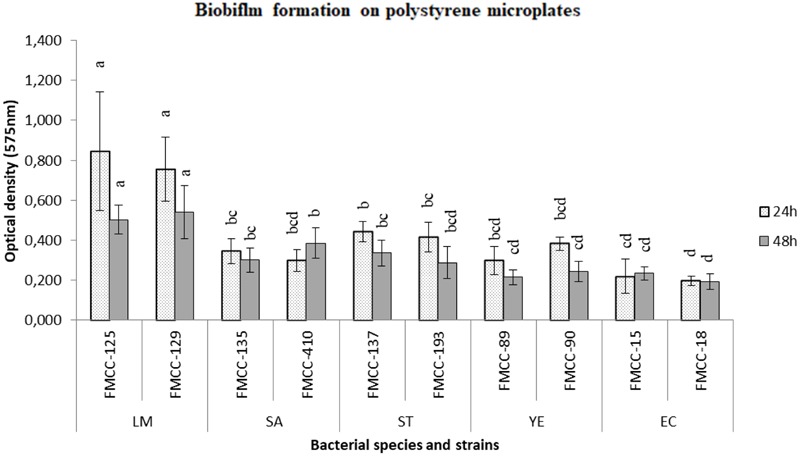
Biofilm formation on polystyrene microtiter plates of different strains after 24 and 48 h of incubation at 37°C. Biofilm cells were indirectly quantified by crystal violet staining and absorbance measurements at 575 nm. Bars represent means ± standard deviations. Different letters at 24 or 48 h indicate significant differences between biofilm formation of strains (*P* < 0.05).

The biofilm forming capacity of five foodborne pathogens at strain level was initially examined in this study by crystal violet method. Briefly, two strains of each pathogen, i.e.,*S.* Typhimurium, *L. monocytogenes, E. coli, Y. enterocolitica*, and *S. aureus* were left to form biofilm on microtiter plate at 37°C to check the strain variability on this phenomenon. In addition, the influence of incubation time, i.e., 24 and 48 h was estimated. The average optical density (OD575) values were calculated for all tested strains at 24 and 48 h (**Figure 1**).

*Listeria monocytogenes* FMCC-125 was classified as strongly biofilm producing strain, while *E. coli* O157:H7 FMCC-16 was evaluated as non-biofilm producer. In addition, both strains of *S. aureus* (FMCC-135, 410), both strains of *Y. enterocolitica* (FMCC 89, 90) and one strain of *E. coli* O157:H7 (FMCC-15) was classified as weak biofilm producers. The rest three strains, consisted of both strains of *S*. Typhimurium (FMCC-137, 193) and a strain of *L. monocytogenes* were classified as moderate biofilm producers.

The previous tested strains were left to form dual strain biofilm on stainless steel and glass surfaces. In accordance to the previous analyzed results, it was observed that biofilm formation was influenced by the bacterial species and incubation time; however, the effect of surface was also estimated. Briefly, a statistical difference was detected between biofilm formation on glass and stainless steel both at attachment step and formed biofilm (24 and 48 h). More specifically, in the case of glass surface the attached and biofilm cell population was found to be lower than on stainless steel surface.

**FIGURE 2:**
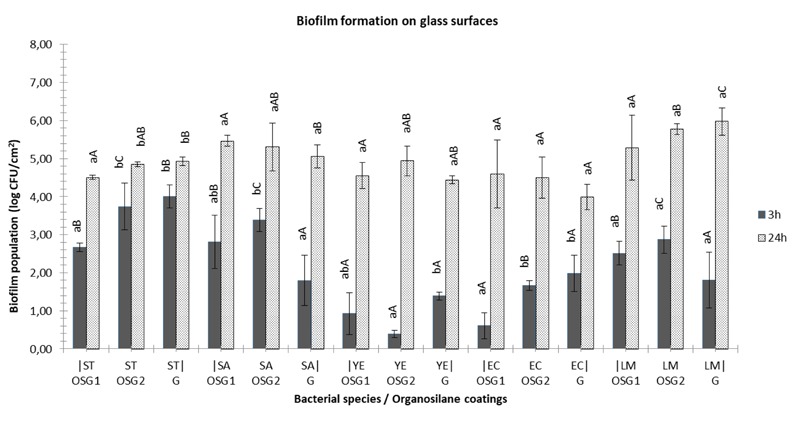
Biofilm formation (log CFU/cm^2^) on glass coupons with (OSG1/OSG2) or without (G) coating, using two strains of *Salmonella* Typhimurium (ST), *Staphylococcus aureus* (SA), *Yersinia enterocolitica* (YE), *Escherichia coli* (EC) or *Listeria monocytogenes* (LM) at 3 h (gray bars) and 24 h (white bars) of incubation at 37°C. Bars represent means ± standard deviations. Different lowercase letters indicate differences on cells attachment (3 h) or biofilm formation (24 h) according to coating for the same species. Similarly, different uppercase letters point out differences according to species adherence/biofilm formation for the same coated or non-surfaces.

Regarding the observations related to the attachment ability of the pathogens on non-coated glass surfaces (assessment of the population at 3 h), it seems that *S*. Typhimurium was attached in higher populations (about 4.32 log CFU/cm^2^), while *S. aureus, E. coli, L. monocytogenes*, and *Y. enterocolitica* were attached at significant lower concentrations (1.6–2.7 log CFU/cm^2^) (**Figure 2**; *P* < 0.05). However, *L. monocytogenes* and *E. coli* biofilm population was the highest and lowest, respectively (*P* < 0.05), while *S*. Typhimurium, *S. aureus*, and *Y. enterocolitica* biofilm populations were in similar levels at 24 h. Similar observations reported above regarding the data obtained from the microtiter plates assay. After 48 h, *Y. enterocolitica* biofilm population was significant lower than those of *S.* Typhimurium and *L. monocytogenes* while *S*. Typhimurium and *E. coli* were found to maintain higher level of sessile cells than *Y. enterocolitica*, at 72 h.

Biofilm cycles of *S*. Typhimurium and *S. aureus* had similar trend as they reached the higher biofilm formation at 24 h, while a significant reduction of sessile cells was observed at 72 h. *E. coli* had a different respond as remained throughout incubation period at approximately same numbers of 24 h biofilm population. Lower numbers of cells were retrieved after 48 h of incubation as concern *L. monocytogenes* and *Y. enterocolitica* compared to biofilm formation of 24 h. At 72 h, *L. monocytogenes* sessile cells were remained at levels estimated at 48 h, while a further reduction was observed for *Y. enterocolitica*.

*Staphylococcus aureus* was found to be attached on stainless steel surfaces at a significant higher level compared to glass surfaces. Similar adhesion to glass and stainless steel surfaces and no correlation between materials surface hydrophobicity was obtained for all other species. Biofilm formation at 24 h was found to be significant lower on glass surfaces for *S*. Typhimurium, *Y. enterocolitica*, and *E. coli* (*P* < 0.05).

The application of organosilane products was found to affect the adhesion of the pathogens (estimation of population at 3 h) on glass surfaces, however, their effect influenced by bacterial species (**Figure 2**). More specific, product OSG1 reduced adhesion of *S.* Typhimurium and *E. coli* compared to bare glass surfaces at approximately 1.4 log CFU/cm^2^. On the other hand, product OSG2 was found to induce the attachment of *S. aureus* at the level of 1.8 log CFU/cm^2^ compared to the non-coated glass coupons. However, it seems that the application of both products affected only the first steps of biofilm formation as no significant differences were observed between coated or not glass surfaces after 24, 48, and 72 h.

**FIGURE 3:**
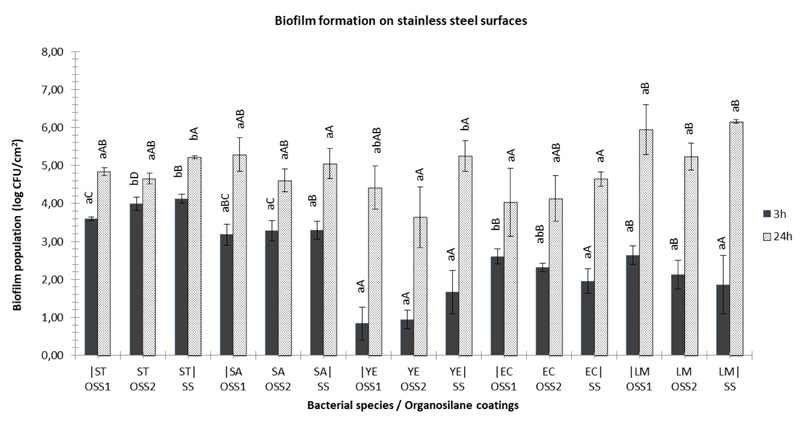
Biofilm formation on glass coupons with (OSS1/OSS2) or without (SS) coating, using two strains of *S*. Typhimurium (ST), *S. aureus* (SA), *Y. enterocolitica* (YE), *E. coli* (EC) or *L. monocytogenes* (LM) at 3 h (gray bars) and 24 h (white bars) of incubation at 37°C. Bars represent means ± standard deviations. Different lowercase letters indicate differences on cells attachment (3 h) or biofilm formation (24 h) according to coating for the same species. Similarly, different uppercase letters point out differences according to species adherence/biofilm formation for the same coated or non-surfaces.

On the other hand, significant differences were detected in the case of organosilanes application on stainless steel surfaces compared to bare ones, which highly depended on the bacterial species and time of incubation (**Figure 3**). Briefly, both OSS1 and OSS2 were able to reduce biofilm formation of *S*. Typhimurium at approximately 0.5 log CFU/cm^2^, at 24 h (*P* < 0.05). Similarly, a reduction of *S. aureus* biofilm cells was observed at the level of 0.8–1.2 log CFU/cm^2^ at 48 h. Biofilm formation of *Y. enterocolitica* was also affected after the application of OSS2 as an approximately 1.8 log CFU/cm^2^ reduction of population was observed at 24 h.

## Discussion

Physicochemical properties of inert substratum and bacterium cell surface are known to have impact on bacterial attachment and biofilm formation, however, the exact correlation with discrete characteristics is difficult as the system is very complex. Hydrophobicity of surfaces has been reported as an important factor affecting the attachment of bacteria on surfaces. Specifically hydrophobicity seems to decrease the adhesion of microorganisms on inert surfaces (van Loosdrecht et al., 1987; Dickson and Daniels, 1991; Bonsaglia et al., 2014) and in the same time increase the detachment of sessile cells (Pereni et al., 2006). Stainless steel is considered a hydrophobic material (Lafuma and Quéré, 2003), while glass a hydrophilic material (Robert et al., 2001). Modification of surfaces with organosilanes usually increases the hydrophobic qualities and low surface free energy of native surfaces (Kregiel and Niedzielska, 2014).

Regarding the present results organosilanes found to eliminate adherence of *S*. Typhimurium and *E. coli* on modified glass surfaces, but this effect was not evident on stainless steel surfaces. A considerable alteration on physical properties of glass surfaces from hydrophilic to hydrophobic may be the reason of the anti-adherent properties observed. In addition, low surface energy chemistry and nano-textured morphology of the coating (homogeneity of the organosilane layer on glass surfaces) could also result in reduced protein adsorption and inhibition of bacterial attachment (Chen et al., 2013).

Significant reductions on biofilm formation (24 and 48 h) were pointed out for *S. aureus, S. Typhimurium*, and *Y. enterocolitica* on modified with organosilanes stainless steel surfaces as compared to their respective controls. A positive correlation between substratum hydrophobicity and the detachment of adherent biofilm was established by other studies. According to this approach, bacteria attached to hydrophobic materials were more easily removed from them (Harkes et al., 1992; Reid et al., 1993; Eginton et al., 1995; Bos et al., 2000; Gómez-Suárez et al., 2001). On the other hand, *S. aureus* found to attach more effectively on stainless steel surfaces in comparison with glass ones, while organosilanes enhance the adherence of bacterium to modified glass surfaces. It seems that a correlation between hydrophobicity and the number of attached cells was resulted. Organosilanes had no effect on eliminating *L. monocytogenes* attached cells or biofilm formation. No differences were also observed regarding different non-modified glass or stainless steel surfaces. These results are in agreement with other studies, too. Teixeira et al. (2007) claimed that adhesion of *L. monocytogenes* to abiotic surfaces was not influenced by substratum hydrophobicity and roughness.

Silica nanoparticles have been found to eliminate *Candida albicans* adhesion and surface associated growth (Cousins et al., 2007). Another study found that concentration of silicon dioxide above 1000 ppm was required to achieve antibacterial activity against *Bacillus subtilis* and *E. coli* (Adams et al., 2006). Polyethylene surfaces, following activation by plasma processing and modification with active organosilanes, exhibit anti-adhesive and anti-biofilm properties against *Aeromonas hydrophila* (Kregiel and Niedzielska, 2014). Glass surfaces coated with hydrophobic silane (alkyl functionalized silane) modified silica nanoparticles exhibited inhibition performance against the growth of *E. coli, S. aureus*, and *Deinococcus geothermalis* compared to that of pristine silica nanoparticles (Song et al., 2011). Reduction of *S. aureus* and *P. aeruginosa* adherence on super-hydrophobic surfaces synthesized by fluorinated silica colloids was also demonstrated (Privett et al., 2011). On the other hand, silica nanoparticles against oral pathogenic species of *Streptococcus mutans* had limited antibacterial effects, using minimum inhibitory concentration assay for planktonic growth, in 96-well microplates (Besinis et al., 2014). Evaluation of two organosilane products applied on high-touch surfaces in patient rooms of a health care facility revealed that no significant residual antimicrobial activity was observed (Boyce et al., 2014).

Numerous previous studies have described the ability of aforementioned foodborne pathogens to attach to various surfaces and form biofilms (Joseph et al., 2001; Stepanović et al., 2004; Kim et al., 2008; Dourou et al., 2011; Oniciuc et al., 2016), with this ability to be depended on the interaction between intrinsic and extrinsic factors such as the bacterial cells, the attachment surface and the surrounding environmental conditions (Giaouris et al., 2014). However, most of these previous studies were performed by constructing single-strain biofilms, with obtaining results not to be necessarily representative of the bacterial species as whole. Undoubtedly, bacterial strains, even the ones belonging to the same species, may greatly differ in many phenotypic responses, including biofilm formation, and this variability should be always taken into account (Lianou and Koutsoumanis, 2013). This is the reason why in the present study were selected two different strains for each species to form multi-strain biofilm communities. The observed phenotypic variability in biofilm formation which ranges from strong to non-biofilm formers even at strain level underlies the importance of strain level studies related to survival and spread of bacteria.

## Conclusion

To the best of our knowledge, this is the first study evaluating modification of stainless steel and glass surfaces with organosilane based products in order to investigate anti-adhesion and anti-biofilm potential against foodborne pathogens. In conclusion, the current study was able to demonstrate anti-adhesion and anti-biofilm activity of specific organosilane based products, but this aspect highly depended on the species of pathogens used in this study and time of incubation (3, 24, 48, and 72 h). Further studies are needed to establish the underlying mechanisms regarding the role of organosilane based products modification on various surfaces types and bacterial species. On the other hand, nanomaterials could have a fundamental impact on the food and medicine sector, potentially offering benefits as concerning the battle against biofouling.

However, any potential risks for consumers are still required to be estimated and assessed in order to ensure public health. The risk of certain nanomaterial should be evaluated as concern the application, the use and final disposal (Contado, 2015). Furthermore, the risk of consumer exposure to nanoparticles directly from medical implants or indirectly through possible migration from surfaces to foodstuffs should be evaluated, since a knowledge gap exist with regards to absorbance, metabolism, and elimination of nanoparticles from the human body.

## Author Contributions

EG designed the studies, performed the experiments, and wrote the paper. AD designed the studies, performed the experiments, and wrote the paper. NC designed the studies and wrote the paper. G-JN wrote the paper.

## Conflict of Interest Statement

The authors declare that the research was conducted in the absence of any commercial or financial relationships that could be construed as a potential conflict of interest. The reviewer Dr. AC and handling Editor declared their shared affiliation, and the handling Editor states that the process neverthless met the standards of a fair and objective review.

## Footnotes

1 https://www.liquidglassnanotech.com/

2 http://bfphellas.gr/
